# Malaria, COVID-19 and angiotensin-converting enzyme 2: what does the available population data say?

**DOI:** 10.1098/rsob.210213

**Published:** 2021-10-13

**Authors:** A. De, M. Dash, A. Tiwari, A. Sinha

**Affiliations:** ICMR-National Institute of Malaria Research, New Delhi, India

**Keywords:** malaria, COVID-19, ACE2

## Abstract

The etiopathogenesis of COVID-19 and its differential geographic spread suggest some populations are apparently ‘less affected’ through many host-related factors that involve angiotensin-converting enzyme 2 (ACE2) protein, which is also the entry receptor for SARS-CoV-2. The role of ACE2 has been well studied in COVID-19 but not in the context of malaria and COVID-19. We have previously suggested how malaria might intersect with COVID-19 through *ACE2* mutation and here we evaluate the currently available data that could provide a link between the two diseases. Based on the existing global and Indian data on malaria, COVID-19 and the suggested *ACE2* mutation, the association could not be examined robustly, neither accepting nor refuting the suggested hypothesis. We strongly recommend targeted evaluation of this hypothesis through carefully designed robust molecular epidemiological studies.

## Graphical abstract

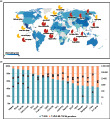

## 1. Background

The novel coronavirus disease COVID-19 that has emerged from Wuhan, China, with the first reported case in December 2019, has ravaged the whole world in such a way that within 3 months, the World Health Organization (WHO) declared it a pandemic. As of 15 July 2021, approximately 18.8 million cases and 4 million deaths have been reported by WHO, globally. Almost every continent has been rapidly infected, the top five worst-hit countries being the USA, Brazil, India, Mexico and Peru, in decreasing order of COVID-19-related deaths [1]. Though the global unadjusted case fatality rate (approx. 2.2%) of SARS-CoV-2 is relatively lower than other fatal corona viruses like MERS (34.4%) and SARS (9.5%) [2,3], its high transmissibility rate (estimated mean basic reproduction number (R_0_) of 2.87) and ability to compromise the respiratory system have made it a major public health concern across the globe [4].

It has been shown convincingly that the spike protein of SARS-CoV-2 interacts with hosts' angiotensin-converting enzyme 2 (ACE2) receptors that are expressed by the epithelial cells of many internal organs like lungs, kidneys and intestine to mediate its entry into the host cell, as earlier reported in other SARS-CoV [5–7]. Thus, the level of ACE2 and its activity play a pivotal role in determining SARS-CoV-2 infections and their downstream clinical profiles. ACE2 is one of the major components of the human renin-angiotensin system (RAS) that counteracts the activity of angiotensin-converting enzyme (ACE) by converting Angiotensin II (AngII) to Angiotensin 1–7 (Ang(1–7)), thus regulating the blood pressure by dilating blood vessels in a healthy adult under physiological conditions [8,9]. The importance of ACE2 lies in the fact that it is a critical regulator of the effector octapeptide levels in human blood, Ang II, the higher levels of which, in turn, contribute to many important pathologies including cardiovascular diseases (hypertension and stroke), diabetes mellitus and lung injury [10–13]. Apart from certain *ACE2* genetic variants, insertion–deletion polymorphism in *ACE* (*ACE I/D*) may also modulate COVID-19 disease severity by elevating physiological Ang II concentration [14,15] such that the DD genotype of *ACE I/D* polymorphism increases the risk of COVID-19 severity [16]. It has been previously shown that an insertion of 287 bp Alu element in intron 16 of the ACE gene is associated with reduced ACE plasma level [17] by inhibiting the RNA polymerase II responsible for ACE mRNA expression [18] or by alternative splicing that results in a truncated ACE protein, thereby losing one of its active sites [19].

The role of elevated Ang II in malaria is hypothesized to offer protection against severe malaria both by killing the parasite and offering haemodynamic stability [20]. In this context, few RAS polymorphisms in *ACE* and *ACE2* have been interrogated [21].

It is to be noted that human ACE2 appears in two forms: membrane-bound ACE2 (mACE2) and circulating ACE2 (cACE2), which is enzymatically shed from mACE2. Apart from mACE2, cACE2 is also shown to retain catalytic activities and the SARS-CoV-2 binding site [22]. Since cACE2 is generated from mACE2, the levels of cACE2 are dependent on the quantitative expression of mACE2 [23].

The entry of SARS-CoV-2 in host cells through the peptidase domain of ACE2 [24,25] appears to downregulate ACE2 [26–28], which could explain the basis of SARS-CoV-2-induced lung injury and ensuing respiratory distress [29,30]. On the other hand, many factors have been implicated to increase the ACE2 expression and/or plasma ACE2 level. Most controversial among these is the use of ACE inhibitors (ACEi) and angiotensin II type-I receptor blockers (ARBs), for example, in hypertension [31–33]. Other factors which are shown to be associated with increased ACE2 in humans include cardiovascular diseases such as hypertension [34], type 2 diabetes mellitus [35,36], smoking/history of smoking [37], age greater than 60 years [38], male gender [39] and administration of thiazolidinediones and ibuprofen [40]. It could be reasonably hypothesized that conditions leading to an upregulation of ACE2 might increase the accessibility of hosts’ receptor for SARS-CoV-2 to interact and invade thus leading to a higher viral load, pathogenesis and severity.

Besides the above medico-social factors, it has also been suggested in various studies that different genetic variants of *ACE2* show differential expression of *ACE2* in humans [41,42], highlighting the possibilities that individuals with certain *ACE2* polymorphisms that lead to an increased overall ACE2 expression might be inherently put at a higher risk of SARS-CoV-2 infection and/or severity, compared to those with no such polymorphisms.

Studies that linked *ACE2* polymorphisms with diabetes mellitus, stroke and hypertension, either singly or together [10–13], specifically in Asian populations [40,43], partially explain the higher risk of COVID-19-related severity. Further, a recent seminal research established that cACE2 levels, in addition to mACE2, mediate the SARS-CoV-2 entry into host cells, either singly or in association with the plasma vasopressin levels through AT1 (Ang II type 1) or AVPR1B (arginine vasopressin receptor 1B) receptors, respectively [22]. This further adds substance to the current hypothesis that reduced mACE2 would lead to reduced cACE2 and since both these forms of ACE2 could be the entry points of SARS-CoV-2, and would lead to a reduced infection.

On a different note, it has already been strongly hypothesized that the T-allele of *ACE2* rs2106809 (as opposed to its C-allele) decreases the ACE2 expression/activity and the ensuing increased AngII forms the basis for protection against severe malaria on one hand but predisposes to hypertension on the other [21,44–46]. Importantly, the hypothesized crucial link between malaria-protective polymorphism (T-allele of *ACE2* rs2106809) and SARS-CoV-2 infection/severity might involve different complex and counteractive mechanisms [44]. The interpretations from these findings may be far more complex than they appear owing to the intricacies inherently involved in RAS [47], scarcity of robust and dedicated studies, and incomplete understanding of regulatory mechanisms. However, recent studies suggested that *ACE2* rs2106809 (T-allele) might reduce cACE2 by downregulating mACE2 gene expression and/or promoting its translational repression and/or posttranscriptional degradation [41,48–51]. Recently, this hypothesis has been tested through gene-tissue expression (GTEx) analyses of most significant eQTLs that contribute to ACE2 expression and convincingly shown that the genetic variant rs2106809 T-allele has significantly lower ACE2 expression, globally [52]. This study also depicts an inverse relation between ACE2 expression and COVID-19 fatality, both at population and molecular levels, supporting the proposed hypothesis [53].

Since such mutations in human *ACE2* are implicated in providing protection from severe malaria in regions that are currently malaria-endemic or have been chronically exposed to severe malaria, there is a strong proposition that such severe-malaria-protective polymorphisms have been positively selected over time. Hence, it is believed that a population that has been living in malaria-endemic areas will have a higher proportion of compensatory genetic variant (T-allele of *ACE2* rs2106809) leading to a lower ACE2 level/activity (protective for SARS-CoV-2 infection) and higher AngII levels (protective for severe/cerebral malaria but augmentative for COVID-19-related pathologies) [44].

**Figure 1.**
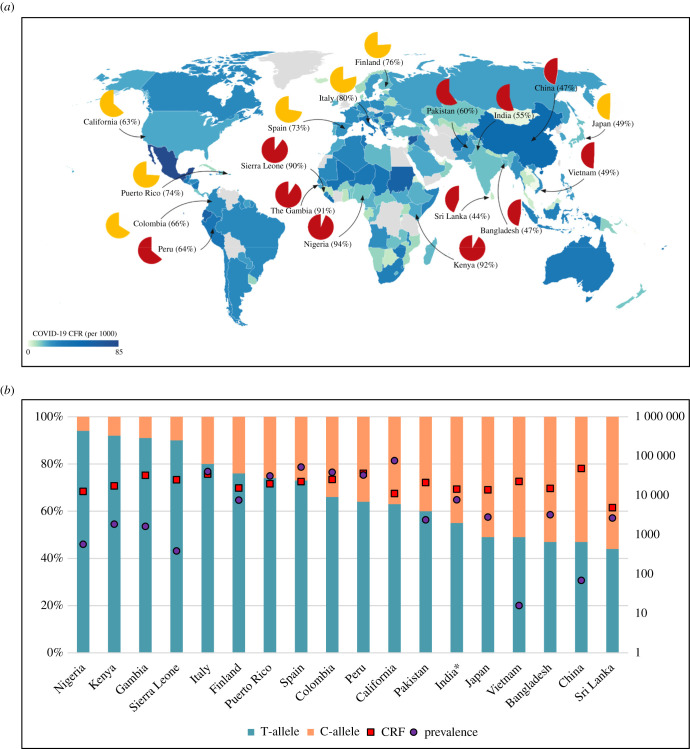
Global COVID-19 and *ACE2* rs2106809 distribution. COVID-19 data were taken from the WHO dashboard, as of 22 January 2021 (https://covid19.who.int/table). *ACE2* rs2106809 data were taken from the 1000 Genomes Project [54]. The *ACE2* rs2106809 data were excluded where their respective country representativeness was not clear. (*a*) Choropleth map showing COVID-19 case fatality rate (CFR; deaths per 1000 cases) across the globe (grey denotes no COVID-19 CFR data). The open pie charts represent the *ACE2* rs2106809 T-allele percentage distribution, in parenthesis beside the country name, according to malaria endemicity (in terms of countries exposed to malaria in the last 30 years); maroon and yellow pie charts represent malaria-endemic and non-endemic countries, respectively. The choropleth map was prepared using datawrapper (https://app.datawrapper.de/). (*b*) Stacked bar chart showing the *ACE2* rs2106809 allele percentatge distribution (T/C) in selected countries, with T-allele (turquoise blue) and C-allele (peach) on the left *y*-axis. The countries have been arranged in decreasing order of the proportion of T-allele. The *x*-axis represents selected countries of the population sampled in the 1000 Genomes Project [54]. COVID-19 related parameters are shown in log-scale on the left *y*-axis, with CFR (red square; per million cases) and prevalence (violet circle; per million population). The country-specific population data were derived from United Nations, Department of Economic and Social Affairs 2017 (https://population.un.org/wpp/Publications/Files/WPP2017_KeyFindings.pdf). *Note: The populations sampled for India were not living in India, but had an Indian origin (Gujarati Indians in Houston, Texas, USA and Indian Telugu in the UK).

**Figure 2.**
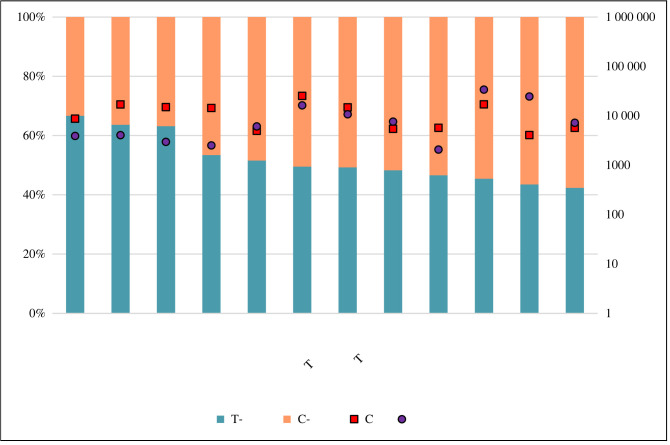
Stacked bar chart showing the *ACE2* rs2106809 allele percentage distribution (T/C) in selected Indian states (with sample size more than 30) from the IndiGenomes database, with T-allele (turquoise blue) and C-allele (peach) along left *y*-axis. The states have been arranged in the decreasing order of proportion of T-allele. The *x*-axis shows the selected states from where the populations were sampled in the IndiGenomes database [56]. COVID-19-related parameters are shown in log-scale on the right *y*-axis, with CFR (red square; per million cases) and prevalence (violet circle; per million population). The COVID-19 data were retrieved from India government COVID-19 dashboard as on 22 January 2021 (https://www.mygov.in/corona-data/covid19-statewise-status/). The state-specific population information was retrieved from the Unique Identification Authority of India as on 31 December 2020 (https://uidai.gov.in/images/state-wise-aadhaar-saturation.pdf). Allele frequencies were calculated from the IndiGenomes genotype data using Hardy–Weinberg equilibrium equation.

In order to be able to comprehend the potential association of *ACE2* rs2106809 T-allele with COVID-19 epidemiology, the distribution of population bearing the T-allele (TT/T genotype) was analysed along with COVID-19 morbidity (cases per million population) and mortality (case fatality rate per million COVID-19 cases), as shown in figure 1 (see also electronic supplementary material). The worldwide data for COVID-19 were taken from the WHO dashboard, as of 22 January 2021 (https://covid19.who.int/table). The global allele frequency data of *ACE2* rs2106809 was retrieved from the 1000 Genomes Project [49] and state-specific distribution of *ACE2* rs2106809 in India was retrieved from the IndiGenomes database (http://clingen.igib.res.in/indigen/). As per the data from the 1000 Genomes Project [54], it appears that the African population has the highest proportion of *ACE2* rs2106809 T-allele (greater than 80%), which is explainable by its strong positive selection due to hypothesized protection from malaria [21,46,55] (figure 1). If the hypothesis to be believed, the same should hold true for the South-east Asian population (*ACE2* rs2106809 T-allele 40–60%), but due to the nature of sampling in the 1000 Genomes Project for the Indian population [54], the available data could not be conclusive, as discussed below. The European population showing a high proportion of *ACE2* rs2106809 T-allele (70–80%) is quite surprising, although it might be hypothesized that the T-allele might be selected due to protection offered to other diseases as well (unconfirmed). In addition, when the IndiGenomes data [56] were analysed for the same allele across samples from India, no significant pattern of T-allele distribution was observed that could explain the selection of this allele in the ‘classical’ higher 5-year (2016–2020) average annual parasite incidence (API) in malaria-endemic areas, with the state of Odisha (API 28 per 1000 population) showing the lower proportion of T-allele (approx. 40%) as compared to the state of Rajasthan (API 0.75 per 1000 population; T-allele proportion greater than 67%) (figure 2).

When the COVID-19 case burden was superimposed on the available *ACE2* rs2106809 T-allele data, it is evident that the case burden was in the lower range in African countries (Nigeria, Kenya, Gambia and Sierra Leone), supporting the hypothesis that the T-allele of *ACE2* rs2106809 might protect against SARS-CoV-2 infection. However, this apparently protective association becomes inconspicuous as we move further to the countries with lower T-allele proportions that were found to have a COVID-19 case burden even lower than in the African countries, for example, Vietnam and China. Further, what might have happened to COVID-19 pathogenesis in Europe and Japan could be explained by the fact that Europe has the second-highest proportion of persons aged more than 70 years in the world after Japan (Japan 18.49%, Italy 16.24%, Spain 14.8%), averaging approximately 15% as compared to a little over 1% in Africa [57]. In addition, it may be plausible that the protective benefit offered by the T-allele is over-compensated by the comorbidities it predisposes to, especially hypertension, whose treatment (ACEi/ARBs) is hypothesized to increase ACE2 expression/levels.

The association of hypertension and COVID-19 might be examined not only in terms of the prevalence of hypertension, which is slightly higher in Central and Eastern Europe as compared to Sub-Saharan Africa [58–60], but also in terms of the pathophysiology of hypertension and the proportion of hypertensive patients taking treatment. A disparity has been documented both in the pathophysiology of hypertension (the hypertension ‘phenotype’) between Africans and Europeans [61], and the percentage of hypertensive population taking anti-hypertensive treatment [60]. Europeans have a ‘high-renin’ hypertension phenotype [61] (which is suggested to have a higher RAS sensitivity) and the proportion of those taking anti-hypertensive treatment is almost twice as much high-income countries as compared to those in the lower-middle-income countries [58,62]. Both these conditions might explain, at least partially, why the COVID-19 associated morbidity and mortality has a disproportionately higher magnitude in Europe and Japan in comparison to Africa despite a reasonably high proportion of rs2106809 TT/T genotype in the population. However, it is very pertinent here to note that individual-level data on both the exposure (*ACE2* rs2106809) and outcome (SARS-CoV-2 infection/COVID-19 pathology) are currently not available, and this is the most relevant factor in examining this plausible association. As per our best knowledge, two studies have attempted to address certain *ACE2* polymorphisms in the context of malaria and COVID-19. Whereas Rusmini *et al.* [63] selected Italian population data to analyse *ACE2* with COVID-19 infection/severity in the context of malaria based on the malaria-protective role of *ACE2*, but the choice of *ACE2* intronic variants (rs4646120 and rs1978124) did not include the ones which have been hypothesized to protect against severe malaria (rs2106809 and its tag-SNPs). However, the variants analysed are reported to decrease the ACE2 expression via increased PIR gene expression thus producing the same ultimate effect of decreasing the ACE2 levels with an associated raised Ang II (in line with our proposed hypothesis). They found that these ACE2-lowering intronic SNPs were negatively associated with the incidence/severity of SARS-CoV-2. Another study that investigated the association of rs2106809-tag SNP rs2285666 (malaria-protective *ACE2* SNPs) with COVID-19 severity failed to demonstrate an association, possibly due to study design and sample size issues [64]. On the other hand, a Chinese population-based study that investigated the association of rs2106809 and SARS susceptibility suggested a statistically protective (OR = 0.9) role of the *ACE2* variant [65]. In summary, no targeted robust studies have been reported that tested this hypothesis rigorously, emphasizing the need to carry out such studies on a priority.

### 1.1. Limitations

Although scientifically explainable, the association between SARS-CoV-2 infection and *ACE2* rs2106809, as inferred from the available data, could be distorted by various factors related to the way *ACE2* mutation data is collected and also by the presence of COVID-19 comorbidities and other host conditions. It is noteworthy that the data on *ACE2* mutation included here are retrieved from two entirely different resources (the 1000 Genomes Project and IndiGenomes), both of which were non-targeted for detecting specific associations.

In addition, both the databases might have lacked external (in terms of geographical and genealogical representativeness of respective countries and populations) and internal (in terms of optimal sample size examined and sufficient statistical power) validities. However, a recent analysis of the laboratory-confirmed COVID-19 hospitalized patients in 14 US states suggests that African-American/black populations in America and males are disproportionately affected by COVID-19, which further suggests the possible effects of the race (African descent with probable malaria exposures) and gender on COVID-19 susceptibilities [66]. This is a point of debate and also an indication towards a biased healthcare system, higher rates of comorbidities and occupational hazards, as well as poverty. Moreover, there are no sufficient data available as of now on the genealogy of African-Americans to conclude any racial disparity in terms of genetic variants that make a population more susceptible or protected.

Further, rs2106809 is definitely not the sole genetic determinant of SARS-CoV-2 infection and/or severity/mortality. It may be supplemented with other tag-SNPs within RAS or even RAS-independent genetic determinants of COVID-19. To make interpretation even more complex, there are various other observed determinants for novel coronavirus infection, disease and mortality. Some of these include older (greater than 60 years) age, male gender, presence of other comorbidities like diabetes and hypertension, treatment with RAS blockers (ACEi and ARBs) and ibuprofen. A further layer of intricacy is added by the epidemiology of the disease. On the one hand, since the COVID-19 pandemic is still differentially evolving in different parts of the world, all the countries under analysis may not be in the same phase of epidemic progression. This might significantly affect parameters such as total reported cases as the countries that have reached the peak of epidemic have different case acceleration as compared to the countries which are on either side of the epidemic curve. Other country-specific surveillance-related parameters like differences in case (and recovery) definitions (symptomatic or asymptomatic), SARS-CoV-2 testing strategies (high-risk or symptomatic or mass screening) and platforms (nucleic acid- or antibody-based tests), number (more population per million tested) and scope of laboratory testing (restricted to government or expanded to non-government; chargeable or free testing), nature (forced or voluntary quarantine/isolation, complete or partial lockdown) and timing of intervention (early or late during the epidemic) will ultimately affect the number of cases being reported by a country and hence its comparability across the countries. Apart from the aforementioned limitations, the importance of this study is to encourage researchers to design robust case-control studies in the population affected through COVID-19 to understand the actual etio-pathogenesis to link these two diseases.

## 2. The way forward

Although no concrete conclusion could be drawn from the data discussed, it definitely justifies the need to design targeted studies. Based on the current understanding and availability of data, it can only be suggested at this point that the *ACE2* genetic variant with rs2106809 T-allele might get selected in those populations that are/have been under malaria ‘pressure’, thus reducing the risk of SARS-CoV infection, and if infected due to any other comorbidities they may have more chance of developing severity owing to the ACE2 and angiotensin imbalance. However, the data retrieved from different resources are diverse and separate, which makes it difficult to reach a definite conclusion.

Attempts to link malaria and COVID-19 pathogenesis have thus far speculated a protective role of malaria exposure in either the reduced risk of infection and/or severity of SARS-CoV-2 disease based on non-specific immunomodulation due to commonly used anti-malaria drug, chloroquine [67], or reduced genetic susceptibility of the malaria-exposed population through yet unknown *ACE2* polymorphisms [68]. Because of the discussed uncertainties and the lack of robust evidence, we strongly opine that the suggested mutant *ACE2*-mediated protection offered by antecedent malaria exposure against SARS-CoV-2 infection and COVID-19 pathogenesis needs to be scrutinized. The best way forward is through powerful case-control studies in populations that have developed a COVID-19-related outcome (recovery/death) and/or in populations undergoing a COVID-19-related process (uninfected/infected and/or symptomatic/asymptomatic) by taking such persons as relative cases and controls, and evaluating the association with their *ACE2* genotypes.

## Data accessibility

The relevant data are accessible through a file in the electronic supplementary material. All data sources are mentioned in the manuscript.
